# Factors Associated with a Low-sodium Diet: The Fourth Korean National Health and Nutrition Examination Survey

**DOI:** 10.4178/epih/e2013005

**Published:** 2013-06-20

**Authors:** Won Joon Lee, Hyeon Chang Kim, Sun Min Oh, Dong Phil Choi, Jaelim Cho, Il Suh

**Affiliations:** Department of Preventive Medicine, Yonsei University College of Medicine, Seoul, Korea.

**Keywords:** Food services, Low-sodium diet, Population surveillance, Republic of Korea, Sodium intake

## Abstract

**OBJECTIVES:**

The low-sodium diet is a known preventive factor for hypertension and cardiovascular disease. Factors associated with low-sodium diets should be identified to reduce sodium intake effectively. This study was conducted to identify factors correlated with a low-sodium diet.

**METHODS:**

This cross-sectional study analyzed data from a total of 14,539 Koreans aged 20 years or older, who participated in the Fourth (2007-2009) Korean National Health and Nutrition Examination Survey. A low-sodium diet was defined as having ≤2,000 mg/day based on 24-hour recalls. Multiple logistic regression models were used to assess sex, age, education, number of family members, household income, occupation, alcohol drinking, total energy intake, frequency of eating out, and hypertension management status for their associations with low-sodium diets.

**RESULTS:**

Among all participants, only 13.9% (n=2,016) had low-sodium diets. In the multivariate analysis, 40-49 years of age, clerical work jobs, higher total energy intake, and frequent eating out were inversely associated with low-sodium diets. And female sex and living-alone were associated with low-sodium diets. Lower frequency of eating out was significantly associated with low-sodium diets, even after adjusting for total energy intake and other potential confounders. Adjusted odds ratios (95% confidence interval) for a low-sodium diet were 1.97 (1.49-2.61), 1.47 (1.13-1.91), 1.24 (0.96-1.61), and 1.00 (reference) in people who eat out <1 time/month, 1-3 times/month, 1-6 times/week, and ≥1 time/day, respectively.

**CONCLUSIONS:**

Our study suggests that sex, age, number of family members, occupation, total energy intake, and lower frequency of eating out were associated with a low-sodium diet in Korean adults.

## INTRODUCTION

Societies with a low-sodium diet have been observed to have a relatively low prevalence of hypertension [1], and dietary sodium reduction could reduce blood pressure in not only hypertensive but also normotensive and prehypertensive individuals [2]. Prospective studies investigating the effects of sodium intake on cardiovascular mortality have shown positive associations between dietary sodium intake and the risk of stroke [3,4] and coronary heart disease [5,6]. Also, a modest reduction in dietary salt could substantially reduce cardiovascular events and medical costs [7]. The Lancet Non-Communicable Disease (NCD) Action Group and the NCD Alliance have proposed five priority interventions, each chosen for their health effects, cost-effectiveness, low cost of implementation, and political and financial feasibility. One of these interventions is a reduced salt intake [8].

In Korea, the average sodium intake has been estimated at 4,878 mg/day [9], more than 2.4 times the recommended amount [10]. Previous studies regarding sodium intake in Korea have addressed the sources of sodium intake by looking at sodium levels in prepared dishes [11-14]. However, few studies have evaluated individual factors related to a low-sodium diet. In order to effectively reduce sodium intake in the Korean population, we need to identify factors associated with a low-sodium diet. Accordingly, this study was conducted to identify factors correlated with a low-sodium diet in Korean adults.

## MATERIALS AND METHODS

The Fourth Korean National Health and Nutrition Examination Survey, conducted by the Korea Centers for Disease Control and Prevention over a three-year period (2007-2009), used a complex multistage sampling design to obtain a representative sample of the civilian, non-institutionalized Korean population. A total of 17,240 Koreans aged 20 years or older were enrolled during the 2007-2009 period. Of these, 2,701 participants were excluded from the present study due to absence of variables, and implausible dietary consumption, i.e., <800 kcal/day or >4,000 kcal/day in males, and <500 kcal/day or >3,500 kcal/day in females [15]. In total, 14,539 participants were eligible for this study. All participants provided written informed consent and this study was approved by an institutional review board.

The survey consisted of three components: the Health Interview Survey, the Health Examination Survey and the Nutrition Survey. Data from the Health Interview Survey were used to obtain information on age, education, marital status, number of family members, household income quartiles, occupation, alcohol drinking, hypertension diagnosis, hypertension treatment, and comorbidities, such as stroke, myocardial infarction, ischemic heart disease, and chronic renal failure. Data on blood pressure were obtained from the Health Examination Survey. The frequency of eating out, total energy intake, and dietary sodium intake were obtained from the Nutrition Survey.

Hypertension management status was categorized into five groups: normotension (diastolic blood pressure<80 mmHg and systolic blood pressure<120 mmHg), prehypertension (not included in the normal or hypertensive criteria), hypertension without treatment, hypertension with treatment, and hypertension with comorbidities as listed above. Dietary sodium intake (mg/day) was estimated based on a 24-hour dietary recall, and we defined a low-sodium diet as less than 2,000 mg/day [16].

Thus, age, education, marital status, number of family members, household income, occupation, total energy intake, alcohol drinking, frequency of eating out, and hypertension management status were considered the potential factors associated with a low-sodium intake. Selection of covariates was based on the published literature review and univariate logistic regression analyses. Marital status was excluded because of its lack of statistical significance (p=0.45).

Statistical analyses were performed using SAS version 9.2 (SAS Institute Inc., Cary, NC, USA), accounting for the multistage, stratified survey design, and survey weightings to obtain all estimated coefficients. For the descriptive analyses, gender-specific histograms of sodium intake distributions were created. Categorical variables were described by the number of participants, percentage of participants, estimated population size, and estimated percentage with survey analysis (PROC SURVEYFREQ, and PROC SURVEYMEANS). The mean sodium intake according to the variables was likewise estimated. Univariate logistic regression analysis was used to obtain odds ratios (ORs) and 95% confidence intervals (CIs) for the association between a low-sodium diet and the independent variables. Those factors identified as statistically significant by univariate analysis (p<0.05) were included as independent variables in the multivariate logistic regression model. In order to evaluate the residual confounding effect of total energy intake, the association between the frequency of eating out and a low-sodium diet was further analyzed separately according to the tertiles of total energy intake.

## RESULTS

The characteristics of the study population are shown in Table 1. The mean age of the study population was 45.7 years. Those who were unemployed represented the highest frequency in the occupation variable, explained by the fact that the unemployed group included students and housewives. In total, 13.9% of the study participants consumed less than 2,000 mg/day sodium. This value compares with 11.9% (estimated number of people=3,559,822) in the Korean population.

**Figure 1:**
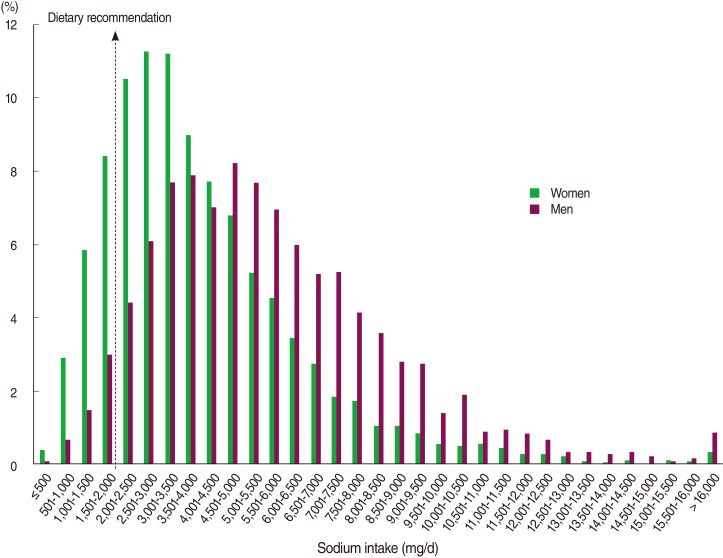
Estimated distribution of sodium intake in Korean women and men.

Figure 1 gives the distribution of sodium intake in Korean women and men. The median (25th-75th percentile) values of sodium intake were 4,208 (2,812-6,159) mg/day in women and 5,205 (3,632-7,247) mg/day in men. The distribution of sodium intake was much higher in men than in women (p<0.05). Accordingly, only 5.2% of male adults (estimated number=706,909) consumed less than 2,000 mg/day sodium, while the corresponding percentage in female adults was 17.5% (estimated number=2,852,912).

Table 2 shows the mean sodium intake and its 95% CI according to the variables examined. In each category, there were significant differences in mean sodium intake according to the statistical test used, such as a t-test or analysis of variance (ANOVA). Mean sodium intake was highest in people aged 40-49 years. Higher levels of education, household income, total energy intake, higher number of family members, alcohol drinking, and more frequent eating out were associated with higher mean sodium intake. According to hypertension management status, those with prehypertension had the highest sodium intake, while those with hypertension with comorbidity consumed the least sodium.

The factors associated with a low-sodium diet are shown in Table 3. All covariates were significantly associated with a low-sodium diet in the univariate logistic regression. However, the associations found with education, household income, alcohol drinking, and hypertension management status disappeared in the multivariate analysis. The following factors were associated with a reduced OR for a low-sodium diet: aged 40-49 years (OR=0.63 compared to 20-39 years), and employed in clerical work (OR=0.56 compared to unemployment). A low-sodium intake was associated with female sex (OR=1.55) and living alone (OR=1.31 compared to living in a large family, i.e., ≥4 people). Total energy intake was the strongest determinant of a low-sodium diet. Therefore, the ORs for a low-sodium diet increased with decreasing total energy intake: >75% (reference), 50-74% (OR=2.58), 25-49% (OR=7.19), and <25% (OR=25.82). People who ate out less than once per month (OR=2.01) and one to three times per month (OR=1.51) had significantly higher odds of having a low-sodium diet when compared with those who ate out more than once per day.

**Figure 2:**
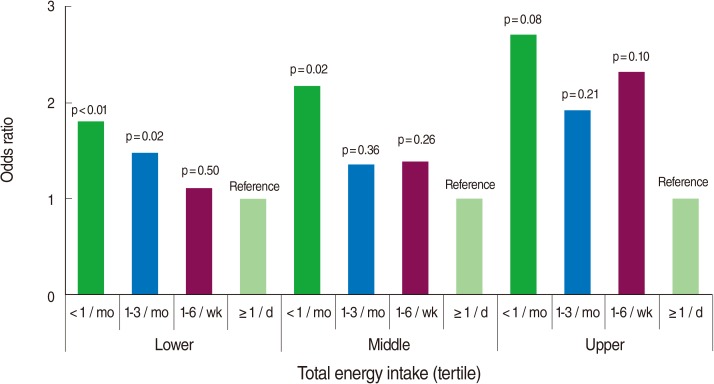
The association between frequency of eating out and low-sodium intake by total energy intake^*^. ^*^Adjusted for sex, age, education, number of family members, household income, occupation, total energy intake (as a continuous variable), alcohol drinking, frequency of eating out, and hypertension management status.

Figure 2 illustrates the association between the frequency of eating out and a low-sodium diet separately in the tertiles of total energy intake. Each model was adjusted for sex, age, education, number of family members, household income, occupation, total energy intake (as a continuous measure), alcohol drinking, and hypertension management status. The OR for a low-sodium diet tended to decrease with increased frequency of eating out, divided by tertile of total energy intake in all three groups, although the statistical significance varied between groups.

## DISCUSSION

Our results indicate that next to total energy intake, frequency of eating out was one of the most important determinants of a low-sodium diet, followed by sex, age, number of family members, and occupation. On the other hand, factors such as education, household income, alcohol drinking, and hypertension management status were not significantly associated with the consumption of a low-sodium diet.

This association between the frequency of eating out and a low-sodium diet was found in Korean adults. This observation is in agreement with a previous study of occupational settings [17]. It implies that interventions aimed at reducing the frequency of eating out may be effective in reducing sodium intake. Individual efforts to reduce the frequency of eating out may help to reduce sodium intake. In the meantime, policies concerning the food service industry could effectively reduce sodium intake at the population level.

Individuals in a higher income bracket have been shown to have a significantly higher mean intake for most of the nutrients examined, including total energy and sodium [18]. The current study similarly showed that having a higher household income was associated with a lower probability for a low-sodium diet. However, after adjusting for total energy intake, the relationship between income and a low-sodium diet disappeared.

A low-sodium diet was more frequent in those who lived alone compared with those who lived in a large family (≥4 people). A larger family is more likely to include a senior (elderly) family member. It is known that the elderly prefer a salty taste because the senses of taste decrease with normal aging and disease [19]. Consequently, people living in a large family might tend to consume more sodium.

We expected that people who were under treatment for hypertension and those who had hypertension-related cormobidities would consume less sodium than those who were normotensive. However, unexpectedly, there was no association between hypertension management status and a low-sodium diet in the multivariate analysis. The majority of people consumed more than the recommended amount of sodium, regardless of their blood pressure levels and hypertension-related health problems. One of the possible reasons for this negative result could be that individual efforts to reduce sodium intake has little effect on sodium intake, because almost all Korean food is high in sodium content. Another reason could be that hypertensive patients, with or without comorbidity, are mainly controlled by the use of hypertensive medication, and both patients and physicians make little effort to change their eating habits [20].

The present study also has several limitations. Firstly, because of its cross-sectional design, this study could not establish a temporal relationship between the factors studied and a low-sodium diet. Secondly, there might be measurement error in the sodium intake which was based on a 24-hour dietary recall. A previous study showed that 24-hour dietary recalls yielded estimates of sodium intake that were about 22% less than those obtained from 24-hour urine collection [21]. Therefore, the mean sodium intake would be underestimated using a 24-hour recall estimate, and the number of people with a low-sodium diet would be overestimated when compared to actual sodium intake. If the measurement error was non-differential, the observed associations between the factors analyzed and a low-sodium diet would likely be weaker than the real associations. It is known that a single 24-hour recall does not represent usual intake because of large day-to-day variations [15]. However, this method has been most widely used in national nutritional surveys such as the National Health and Nutrition Examination Survey, United Sates Department of Agriculture Nationwide Food Consumption Surveys, and the Continuing Surveys of Food Intakes by Individuals.

In conclusion, sex, age, number of family members, occupation, total energy intake, and frequency of eating out were shown to be correlated with a low-sodium diet in Korean adults. Strategies concerning the frequency of eating out or policies regulating the food service industry would be effective in reducing overall sodium intake in Koreans.

## Figures and Tables

**Table 1:**
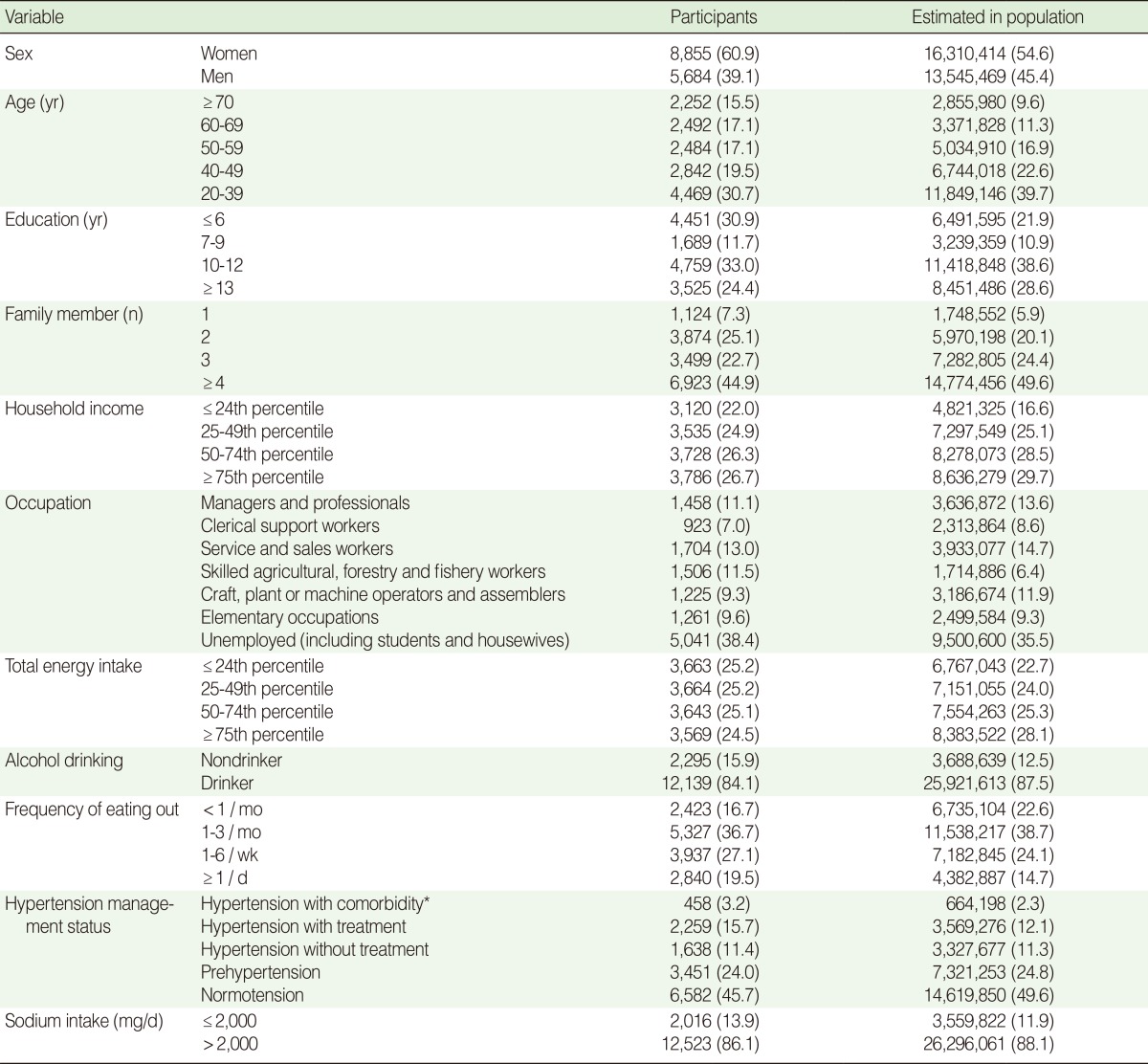
Characteristics of study population (n=14,539)

Values are presented as number (%). ^*^Comorbidity includes stroke, myocardial infarction, ischemic heart disease, and chronic renal failure.

**Table 2:**
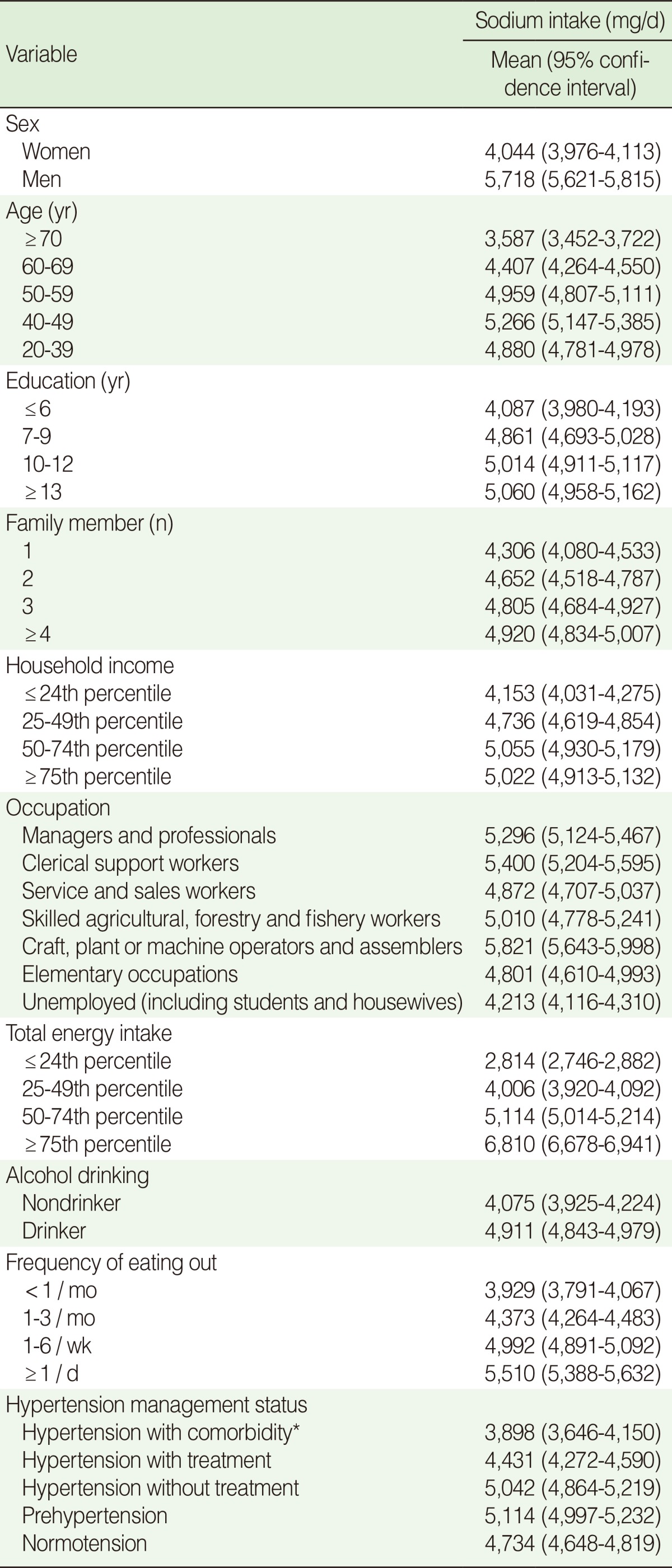
Mean sodium intake according to factors studied

^*^Comorbidity includes stroke, myocardial infarction, ischemic heart disease, and chronic renal failure.

**Table 3:**
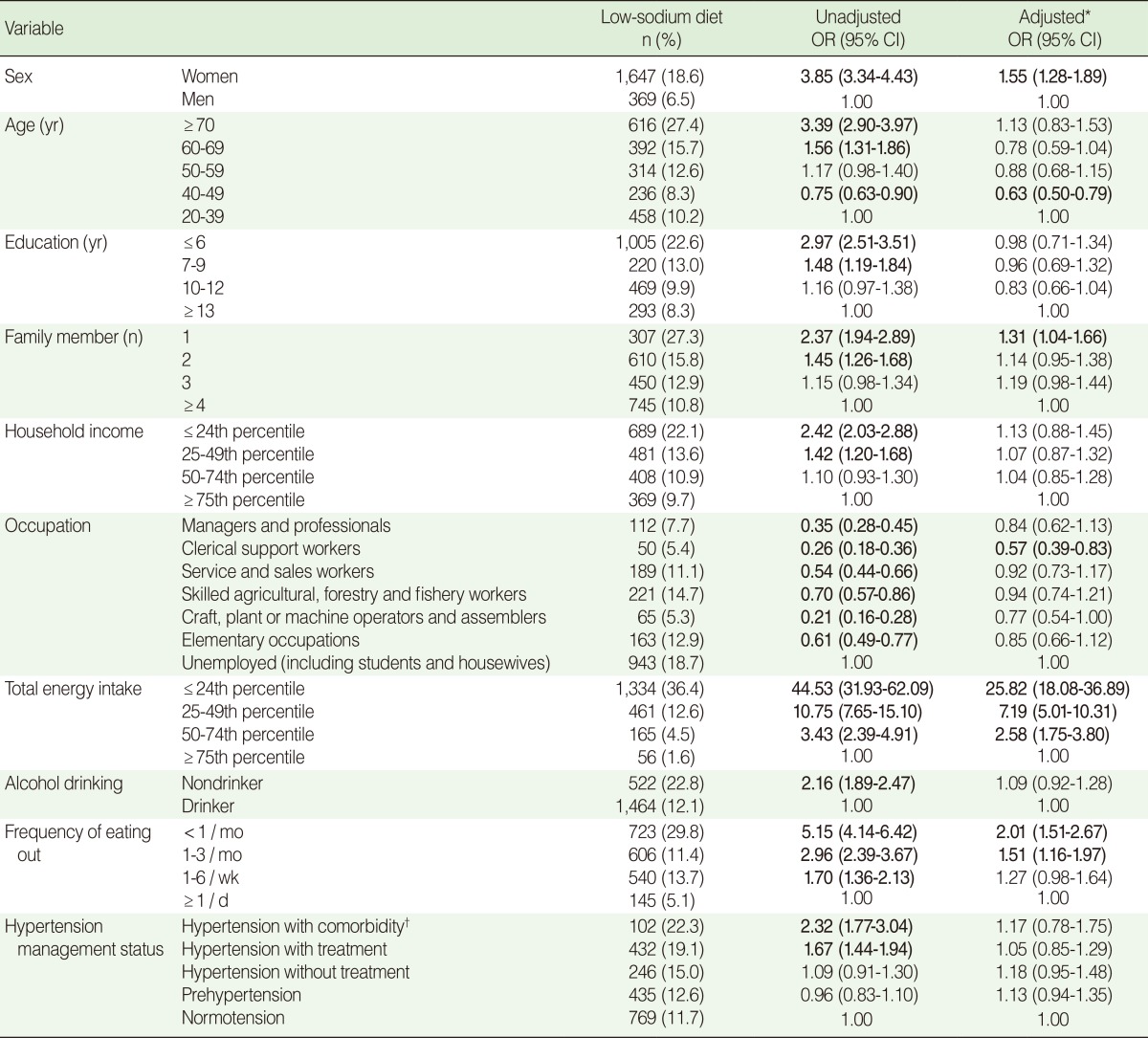
Factors associated with a low-sodium diet (≤ 2,000 mg/day)

OR, odds ratio; CI, confidence interval Statistically significant estimates (p<0.05) appear in bold. ^*^Adjusted for sex, age, education, number of family members, household income, occupation, total energy intake, alcohol drinking, frequency of eating out, and hypertension management status; ^†^Comorbidity includes stroke, myocardial infarction, ischemic heart disease and chronic renal failure.
